# Voluntary Exercise Training: Analysis of Mice in Uninjured, Inflammatory, and Nerve-Injured Pain States

**DOI:** 10.1371/journal.pone.0133191

**Published:** 2015-07-21

**Authors:** Tayler D. Sheahan, Bryan A. Copits, Judith P. Golden, Robert W. Gereau

**Affiliations:** 1 Washington University Pain Center and Department of Anesthesiology, Washington University School of Medicine, St. Louis, Missouri, United States of America; 2 Washington University Program in Neuroscience, Washington University School of Medicine, St. Louis, Missouri, United States of America; University of South California, UNITED STATES

## Abstract

Both clinical and animal studies suggest that exercise may be an effective way to manage inflammatory and neuropathic pain conditions. However, existing animal studies commonly use forced exercise paradigms that incorporate varying degrees of stress, which itself can elicit analgesia, and thus may complicate the interpretation of the effects of exercise on pain. We investigated the analgesic potential of voluntary wheel running in the formalin model of acute inflammatory pain and the spared nerve injury model of neuropathic pain in mice. In uninjured, adult C57BL/6J mice, 1 to 4 weeks of exercise training did not alter nociceptive thresholds, lumbar dorsal root ganglia neuronal excitability, or hindpaw intraepidermal innervation. Further, exercise training failed to attenuate formalin-induced spontaneous pain. Lastly, 2 weeks of exercise training was ineffective in reversing spared nerve injury-induced mechanical hypersensitivity or in improving muscle wasting or hindpaw denervation. These findings indicate that in contrast to rodent forced exercise paradigms, short durations of voluntary wheel running do not improve pain-like symptoms in mouse models of acute inflammation and peripheral nerve injury.

## Introduction

Chronic pain is a debilitating condition that effects over 100 million Americans and has an annual cost of $635 billion in the form of health care expenses and productivity loss [1,2]. Clinical studies suggest aerobic exercise is an effective, non-invasive approach to managing ongoing inflammatory and neuropathic pain conditions, as well as musculoskeletal disorders [3–7]. Similarly, exercise improves pain-like symptoms in rodent inflammatory and neuropathic pain models [8–20].

The effect of exercise on pain has been primarily evaluated using forced exercise paradigms. For example, recent studies have demonstrated that forced treadmill running following peripheral nerve injury reverses nerve injury-induced thermal and mechanical hypersensitivity in rats and mice [9–11,21,22]. Extended swimming has also been shown to both attenuate the development of and reverse nerve injury-induced thermal and mechanical hypersensitivity in rodents, as well as formalin-induced spontaneous pain in rats [8,14,20]. As acknowledged in these studies and others, forced exercise may elicit both acute and chronic stress responses that in turn can produce analgesia [23–28]. Although most forced exercise studies have evaluated nociceptive thresholds after exercise-induced acute stress responses have resolved, prolonged forced exercise gives rise to chronic stress responses in some cases [23,25,26]. Voluntary exercise may also elicit a stress response [24]; however, numerous studies demonstrate anxiolytic effects of prolonged voluntary wheel running in cases of mild to moderate stress [29–33]. One way to minimize potential complications of stress dependent effects on pain is to use voluntary exercise paradigms.

Voluntary wheel running has been shown to be effective in delaying decreases in muscle withdrawal thresholds and increased paw withdrawal frequency in mouse models of chronic muscle pain [13]. Similarly, in a high-fat model of prediabetic neuropathy, voluntary wheel running reverses mechanical and visceral hypersensitivity [17]. While these studies support that voluntary wheel running improves pain-like behavior in rodents, reports utilizing voluntary exercise paradigms in injured and uninjured pain states are limited.

To investigate whether repeated voluntary exercise sessions alter basal nociception in rodents, we determined whether voluntary wheel running changes nociceptive thresholds, sensory neuron excitability, or skin innervation in the absence of injury. We also investigated if voluntary exercise training alters acute inflammatory pain responses as well as the hypersensitivity, muscle wasting, or skin denervation induced by the spared nerve injury (SNI) model of neuropathic pain. We report that voluntary wheel running did not alter nociceptive thresholds in uninjured mice, and was ineffective in attenuating acute inflammatory or nerve-injury induced pain.

## Materials and Methods

### Ethics Statement

The entire study was carried out in accordance to the guidelines of the Washington University in St. Louis Department of Comparative Medicine (DCM). The protocol was approved by the Animal Studies Committee of DCM (Protocol Number: 20130147).

### Animals

Adult C57BL/6J male mice bred in house or obtained from Jackson Labs were housed and cared for in compliance with the Animal Studies Committee of Washington University in St. Louis. Mice were housed on a 12/12 hour (hr) light/dark cycle and had *ad libitum* access to food and water while in their home cages. Behavioral experiments were initiated on 7–10 week old mice. Over the course of testing, injury (if applicable), and exercise training, mice reached a maximum age of 14 weeks. At the conclusion of behavioral testing, mice were sacrificed using a rodent ketamine euthanasia cocktail.

### Exercise paradigm

During exercise training, mice were placed into individual cages with low-profile wireless running wheels (Med Associates Inc.) for either 2 hr (6–8 PM) or 12 hr (7 PM-7 AM) a night, 5–6 nights a week. A sixth night of training was required to maintain exercise states throughout behavioral testing. Distance ran was monitored using software from Med Associates Inc. Control animals were placed into individual cages with a locked running wheel. During 2 hr training sessions, white noise was used to mask external noises. Animals trained overnight were provided food pellets and Hydrogels (ClearH_2_O) to prevent weight loss. Home cage access to running wheels–either unlocked or locked–was not used in our studies to minimize stress associated with the chronic social isolation of single housing [34]. When not exercising, animals were group housed in their home cages.

Exercise training was completed prior to the induction of acute inflammatory pain, which took place the afternoon following the last exercise bout. Specifically, intraplantar formalin was administered 16 hr after completion of 2 hr/night exercise sessions or 4–6 hr after completion of 12 hr/night exercise sessions. In nerve-injury studies, exercise training began 8–10 days after surgery.

### Behavioral studies

All behavioral assays were completed between 8 AM and 5 PM. Briefly, for the Hargreaves, von Frey, and cold plantar assays, mice were acclimated in plexiglass boxes to the testing platform and white noise for at least 2 hr prior to testing until exploratory behavior ceased. For the hot plate and inverted screen tests, mice were acclimated to the testing room in their home cage with white noise for at least 1 hr prior to testing. For all behavioral studies, the experimenter was blinded to training groups.

### Cold plantar assay

Cold sensitivity was measured as previously described [35,36]. Mice were acclimated to either a 3/8” or 1/4” glass plate and a cold probe was made by packing finely crushed dry ice into a modified syringe 1 cm in diameter. The cold probe was applied to the glass beneath the plantar surface of the hindpaw and the time to paw withdrawal was recorded. 5 trials were conducted on each hindpaw, with 7 minutes (min) between trials on opposite paws, and 15 min between trials on the same paw. A cut off latency of 20 seconds (sec) was used to prevent tissue damage. Withdrawal latencies were determined by averaging right and left paw responses.

### Hargreaves

Animals were acclimated on a glass plate held at 30°C (Model 390 Series 8, IITC Life Science Inc.). A radiant heat source was applied to the hindpaw and latency to paw withdrawal was recorded [37]. 5 trials were conducted on each paw, with at least 5 min between testing the opposite paw and at least 10 min between testing the same paw. To avoid tissue damage, a cut off latency of 20 sec was set. Values from both paws were averaged to determine withdrawal latency.

### Hot plate

Prior to testing day, animals were individually acclimated to the hot plate chamber (Model PE34 Series 8, IITC Life Science Inc.) for 15 min. On the day of testing, one experimental trial was conducted in which each animal was placed onto a 55°C plate and the latency to response was recorded. “Response” was defined as the first act directed towards the hindpaw, which was primarily licking or shaking, or escape behavior such as jumping. A cut off latency of 20 sec was used to prevent tissue damage.

### von Frey

Mice were acclimated on an elevated mesh grid and mechanical sensitivity was determined by applying von Frey filaments to the plantar hindpaw according to the up-down method as described previously [38]. Three trials were conducted on each paw with at least 5 min between trials on opposite paws, and 10 min between trials on the same paw. Mechanical paw withdrawal thresholds of uninjured animals were obtained by averaging the 3 trials performed on both paws. Nerve-injured animals were tested on the lateral aspect of the hindpaw at both baseline and post-injury time points, and the average ipsilateral withdrawal thresholds were calculated [39].

### Formalin test

For acute inflammatory pain, mice were acclimated in plexiglass boxes on a glass platform for 1 hr with white noise. Mice were briefly removed from the platform and 10 μL of 2% formalin was injected subcutaneously into the hindpaw. Mice were video recorded for 1 hr after formalin injection and time spent licking and lifting the paw post-injection was scored in 5-min bins offline. To minimize potential acute effects of exercise on behavior [40,41], the formalin test was performed at least 16 hr after the final 2 hr training session, and at least 4 hr after the final overnight training session.

### Inverted screen test

To evaluate muscle strength, mice were allowed to grasp onto a mesh grid. The grid was then inverted until mice were upside-down and the latency to fall recorded, using a cut off latency of 120 sec. Two trials were performed with at least 1 hr of rest allowed between trials [42].

### Dissociating DRG neurons

Mice were sacrificed via live decapitation and lumbar (L) dorsal root ganglia (DRG) 3–5 were dissected from control and exercise trained mice. DRG were incubated in papain (45U) for 20 min, rinsed, and incubated with collagenase (4.5 mg/200 μL) for 20 min. DRG were triturated to dissociate neurons and filtered with a 40 μm mesh filter. The dissociated cells were plated on poly-D-lysine/collagen coated glass coverslips and were tested 12–24 hr after plating.

### Electrophysiology

Whole-cell recordings were made in current clamp on cells 20–30 μm in diameter to increase the likelihood of recording from nociceptive neurons. Fire polished glass pipettes were pulled using a P-97 micropipette puller (Sutter Instrument Company), and open tip resistances ranged from 2.0–7.0 MΩ. Pipettes were filled with internal recording solution consisting of (in mM): 120 K^+^ gluconate, 5 NaCl, 2 MgCl_2_, 0.1 CaCl_2_, 10 HEPES, 1.1 EGTA, 4 Na_2_ATP, 0.4 Na_2_GTP, 15 phosphocreatine; pH = 7.3, 291 mOsm. While recording, cells were continuously perfused with room temperature external solution consisting of (in mM): 145 NaCl, 3 KCl, 2.5 CaCl_2_, 1.2 MgCl_2_, 10 HEPES, 7 glucose, adjusted to pH 7.4 with NaOH. The series resistance of each recording was less than 20 MΩ. Patchmaster software (Heka Instruments Inc.) controlling an EPC10 USB amplifier (Heka Instruments Inc.) was used to record from neurons. Only neurons with a resting membrane potential more negative than -45 mV were included in data analysis.

### Spared nerve injury model

Throughout surgery, mice were maintained under isofluorane anesthesia. A skin incision was made and the biceps femoris muscle was separated to expose the branches of the sciatic nerve. Without manipulating the sural nerve branch, the common peroneal and tibial braches were ligated with 6–0 silk suture and approximately 1 mm of nerve was removed distally [39]. The wound was closed in layers and mice were monitored for signs of distress and allowed to recover post-operatively on a heating pad prior to returning to their home cages. Surgical procedures were approved by the Washington University in St. Louis DCM (Protocol Number: 20130147).

### Gastrocnemius weight

The gastrocnemius muscle was excised by cutting proximally from medial and lateral condyles of the femur and distally at the calcaneal tuberosity [43]. Tissue was promptly weighed after dissection to obtain muscle wet weight.

### Immunohistochemistry and hindpaw innervation

After sacrificing animals with a rodent ketamine euthanasia cocktail, plantar hindpaw skin was excised and immersion fixed for 2–6 hr in 15% picric acid/2% paraformaldehyde at 4°C, washed in PBS, and cryoprotected in 30% sucrose. Hindpaw skin was sectioned at 30 μm perpendicular to the skin’s surface. To stain hindpaw skin, the primary antibodies rabbit anti-BIII tubulin 1:1000 (Covance PRB-435P) and goat anti-rat CGRP 1:400 (AB Serotec 1720–9007) were visualized using donkey anti-rabbit Alexa-fluor 555 1:200 (Invitrogen A31572) and donkey anti-goat Alexa-fluor 488 1:350 (Invitrogen A11055), respectively. Images were captured using an upright epifluorescent microscope (Nikon 80i, CoolSnap ES camera). MetaMorph software (Molecular Devices, LLC) was used to measure dermal-epidermal border length, and labeled intraepidermal fibers were counted and averaged from 5 nonsequential sections per animal. Intraepidermal nerve fiber (IENF) density is reported as number of fibers/100 μm dermal-epidermal border.

### Epidermal thickness

The vertical distance from the dermal-epidermal border to the outermost granular cell layer–visualized via DAPI staining–was measured at 5 separate points at 50 μm intervals to obtain the average thickness of each section from which IENFs were counted.

### Statistical analyses

Data were analyzed using GraphPad Prism software (GraphPad Software, Inc.) and are presented as mean ± SEM. Withdrawal latencies and thresholds are normalized to baseline values obtained prior to exercise training or SNI. Data comparing the effect of exercise training as well as formalin responses over time were analyzed with a Student’s t-Test. A two-way repeated-measures (RM) analysis of variance (ANOVA) was used to analyze the effect of SNI and exercise training on mechanical thresholds. SNI muscle strength, wet weight, innervation, and epidermal thickness were compared to uninjured controls by a one-way ANOVA. In all analyses, a Bonferroni test was used to correct for multiple comparisons.

## Results

### Nociceptive thresholds were unaltered in uninjured, exercise trained animals

To determine whether exercise training alters the nociceptive thresholds of uninjured mice, we gave mice access to running wheels for 2 hr a night, 5–6 nights a week, for 1–4 weeks. There was an average nightly running distance of 1.5 ± 0.1 km across cohorts. We are confident that the control and exercised animals differ solely in exercise training because during running sessions, controls experienced the same environmental enrichment and social isolation as exercised animals, but were unable to run on the locked wheel. In this regard, voluntary wheel running allows for more comprehensive control groups than forced exercise paradigms.

**Fig 1:**
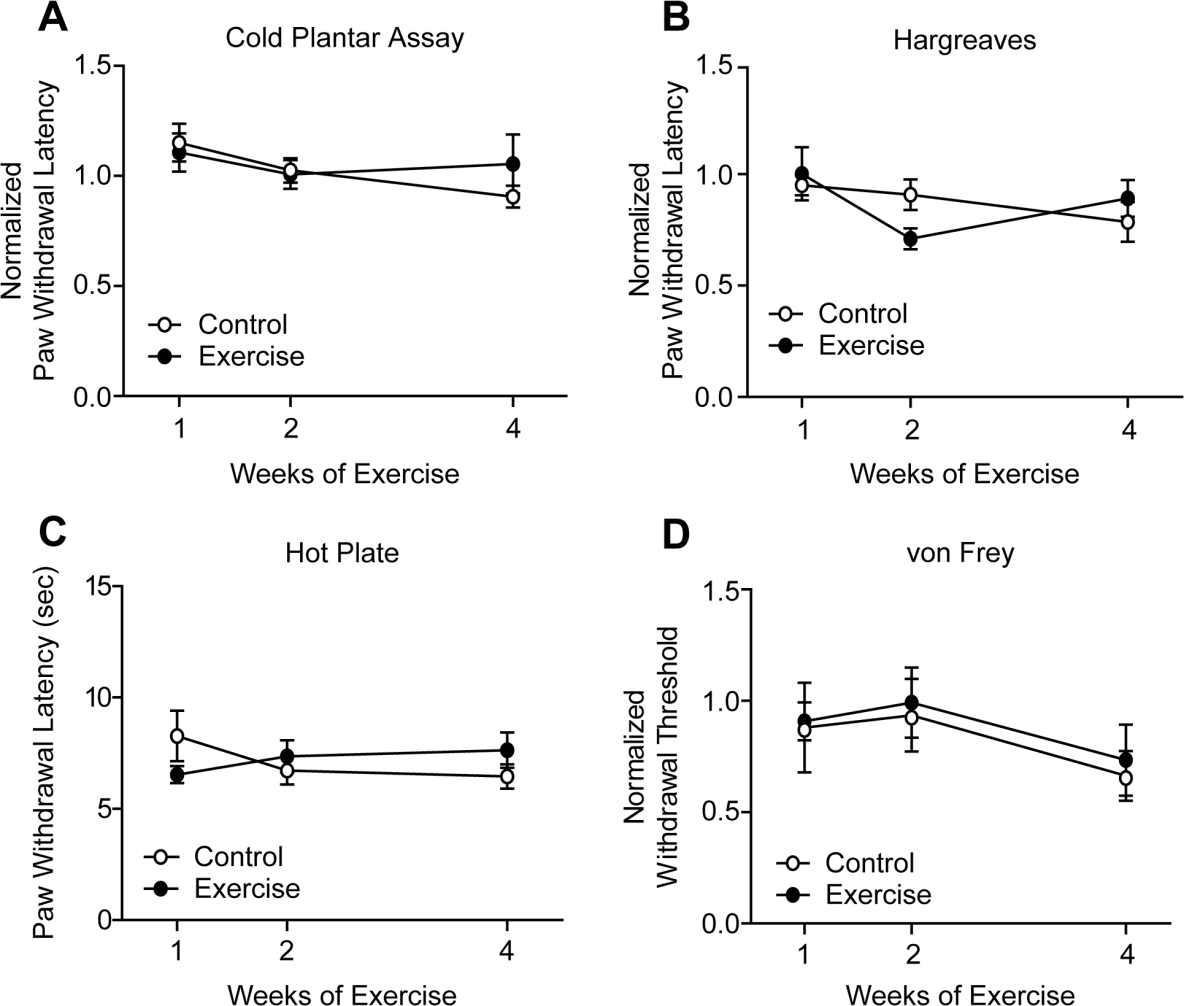
Exercise training did not alter thermal or mechanical sensitivity of uninjured animals. **(A)** Following 1, 2, or 4 weeks of exercise training, paw withdrawal latencies in the cold plantar assay remained unchanged in exercise trained versus control mice, n = 6–11. **(B, C)** Withdrawal latencies to noxious heat stimuli of exercise trained and control mice were equivalent in both Hargreaves and hot plate tests, n = 9–15 and 9–10, respectively. **(D)** Mechanical withdrawal thresholds were similarly unchanged by exercise, n = 9–15. Withdrawal latencies and thresholds are normalized to baseline values obtained prior to exercise training. Data are presented as mean ± SEM. Student’s t-Test, Bonferroni correction for multiple comparisons.

Nociceptive thresholds were measured in uninjured mice at least 12 hr after the conclusion of training sessions to minimize the potential short-term effects of exercise on behavior [40,41]. Following 1, 2, or 4 weeks training, cold sensitivity thresholds of exercised mice measured by the cold plantar assay remained unchanged when compared to controls (Fig 1A). Similarly, noxious heat withdrawal latencies were unchanged in the Hargreaves test after up to 4 weeks of exercise training (Fig 1B). Heat sensitivity was also tested using the hot plate test, which incorporates supraspinal processing of nociceptive stimuli [44,45]. Latency to response to a 55°C hot plate was not different between control and exercise trained mice (Fig 1C). Lastly, exercise training did not alter mechanical withdrawal thresholds in the von Frey test (Fig 1D).

### Sensory neuron excitability was unchanged following exercise training

**Fig 2:**
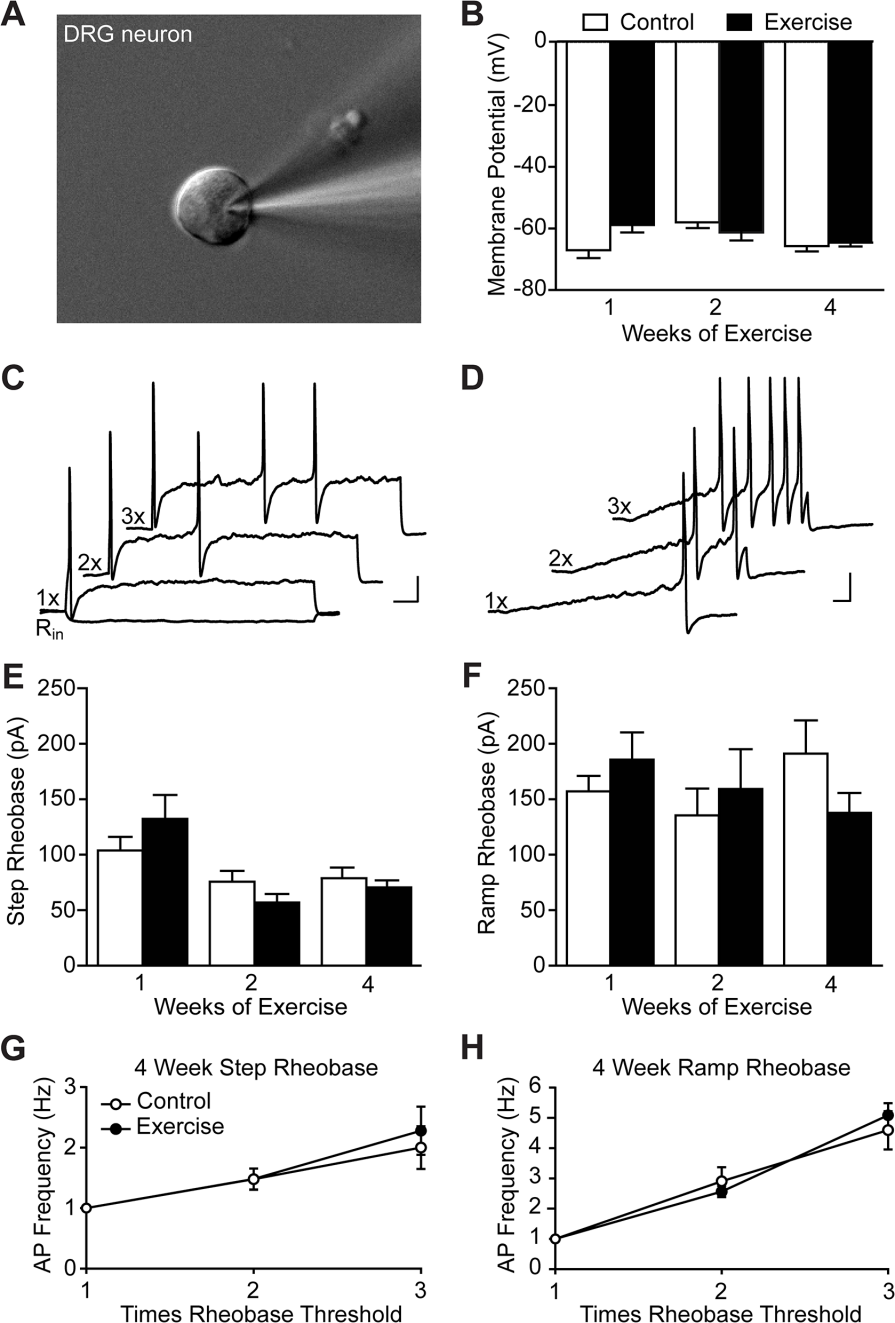
Exercise training did not alter DRG neuron membrane or excitability properties of uninjured animals. Whole-cell patch clamp electrophysiology was performed on small-diameter cultured lumbar DRG neurons, N = 2–3 animals. **(A)** An example of a patched neuron approximately 23 μm in diameter. **(B)** Resting membrane potential was unaltered by exercise training for 1, 2 or 4 weeks, n = 13–25. Representative traces of action potentials elicited by step **(C)** and ramp **(D)** current injections of 1 to 3 times rheobase. A negative stepwise current was used to determine input resistance (R_in_). Scale bars represent 20 mV and 100 ms. Rheobase in response to both step **(E)** and ramp **(F)** current injection was also unchanged by exercise, n = 13–25 and 7–23, respectively. The number of action potentials elicited by step **(G)** and ramp **(H)** current injections 2 and 3 times rheobase was unaffected by 4 weeks of exercise training, n = 22–25 and 20–23, respectively. Data are presented as mean ± SEM. Student’s t-Test, Bonferroni correction for multiple comparisons.

Increased excitability of DRG neurons has been shown to underlie hypersensitivity in a number of pain states [46–48]. To determine whether exercise affects membrane and cell excitability properties in an uninjured context, whole-cell patch clamp electrophysiology was performed on dissociated L3-L5 DRG neuron cultures obtained from exercise trained and control animals within 24 hr of culturing (Fig 2A). Recordings were performed on small diameter neurons ranging from 20–30 μm to increase the likelihood of recording from nociceptive neurons.

Consistent with behavioral experiments, 1–4 weeks of voluntary exercise training did not alter any of the membrane or cell excitability properties analyzed (Table 1). Resting membrane potential was unchanged in exercise trained animals as compared to controls (Fig 2B). In current clamp, step (Fig 2C) and ramp (Fig 2D) current injections were used to analyze excitability properties. Rheobase, the current required to elicit an action potential, in response to either step (Fig 2E) or ramp (Fig 2F) current injections were similarly unaffected by 1–4 weeks of wheel running. The number of action potentials in response to step and ramp current injections 2 and 3 times rheobase were also quantified to investigate if differences in excitability were apparent at higher stimulus intensities. Exercise training for 4 weeks did not change DRG excitability in response to these stimuli (Fig 2G and 2H). Similar results were observed after 1 and 2 weeks of exercise training. Overall, DRG membrane and excitability properties were unaffected by exercise in an uninjured context.

**Table 1:** Membrane and action potential parameters of lumbar DRG neurons from control and exercise trained animals.

Training Duration	1 week		2 weeks		4 weeks	
Parameter	Control	Exercise	Control	Exercise	Control	Exercise
**Cell Diameter, μm**	25.6 ± 0.5	25.2 ± 0.5	23.8 ± 0.7	24.2 ± 0.5	24.6 ± 0.4	24.3 ± 0.3
**Membrane Potential, mV**	-67.1 ± 2.5	-58.9 ± 2.4	-58.0 ± 1.9	-61.6 ± 2.7	-65.7 ± 1.7	-64.6 ± 1.2
**Input Resistance, MΩ**	798 ± 123	608 ± 89	916 ± 133	1396 ± 309	1041 ± 127	936 ± 92
**Capacitance, pF**	18.2 ± 1.0	20.5 ± 1.2	17.2 ± 1.8	18.6 ± 2.2	17.8 ± 1.0	17.8 ± 1.1
**Step Rheobase, pA**	104 ± 12	132 ± 21	76 ± 10	57 ± 8	79 ± 10	70 ± 7
**Step AP Threshold, mV**	-22.7 ± 2.3	-21.2 ± 2.0	-21.0 ±1.8	-23.6 ± 1.2	-22.2 ± 1.3	-20.9 ± 1.9
**Step AP Peak, mV**	49.6 ± 1.2	49.2 ± 1.0	45.0 ± 1.2	47.4 ± 2.0	50.0 ± 1.1	50.4 ± 0.5
**Ramp Rheobase, pA**	157 ± 14	186 ± 24	136 ± 24	159 ± 36	191 ± 30	138 ± 18
**Ramp Threshold, mV**	-11.5 ± 1.6	-14.5 ± 2.2	-12.3 ± 2.9	-16.2 ± 2.3	-13.6 ± 1.6	-13.5 ± 1.6
**Ramp AP Peak, mV**	51.7 ± 1.4	51.3 ± 1.2	44.3 ± 1.6	47.3 ± 2.4	51.3 ± 1.2	50.8 ± 0.6

AP, action potential. Data are presented as mean ± SEM. N = 2–3 animals, n = 7–25 cells. Student’s t-Test, Bonferroni correction for multiple comparisons.

### Acute inflammatory pain responses were not changed by exercise training

**Fig 3:**
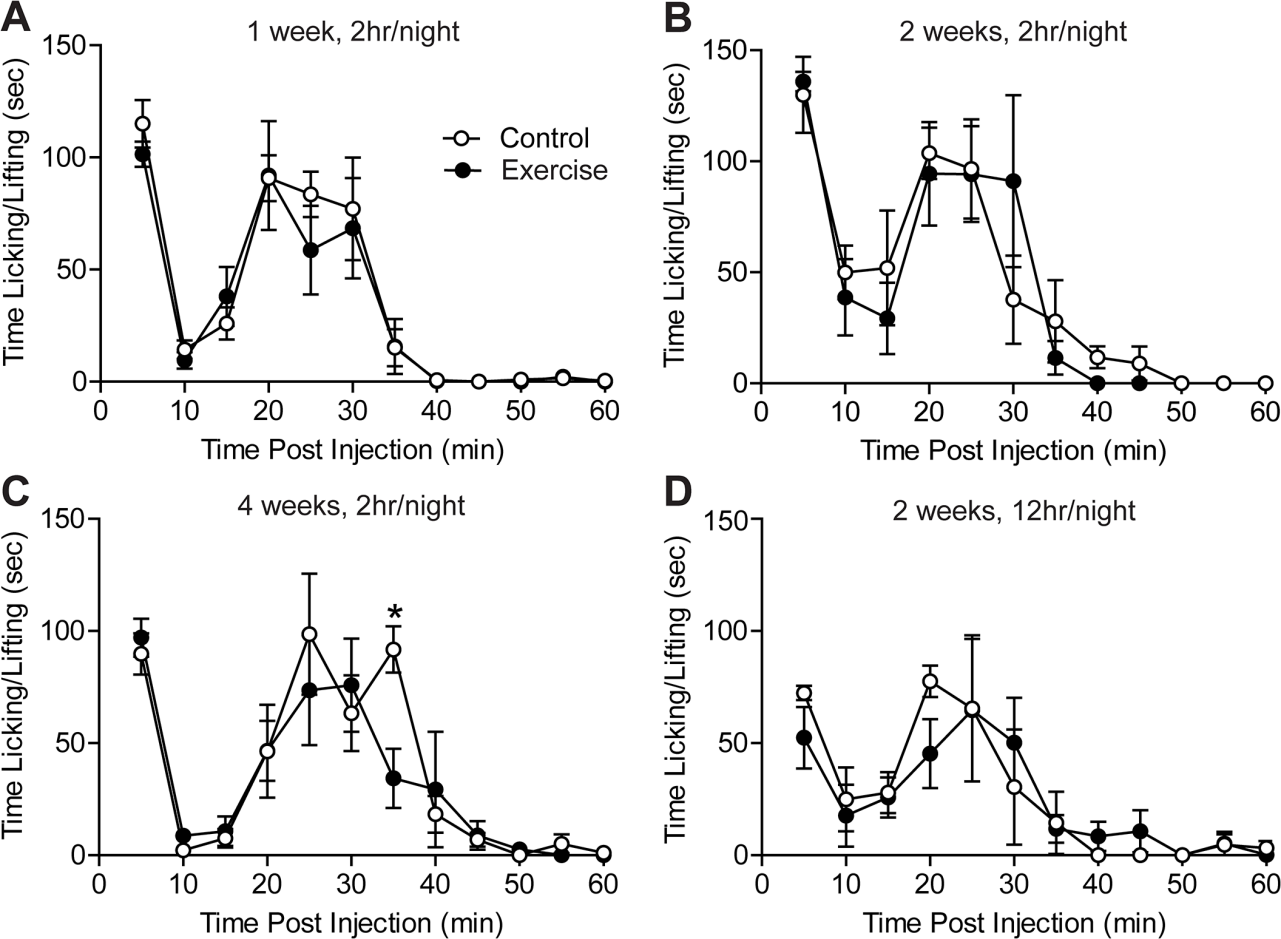
Exercise training did not attenuate nocifensive responses to acute inflammatory pain. **(A-C)** Wheel access for either 2 hr/night for 1 to 4 weeks, n = 5 or **(D)** 12 hr/night for 2 weeks, n = 4–6 prior to the formalin test did not reduce time spent licking or lifting the hindpaw during the first 60 minutes following intraplantar injection of 2% formalin. Data are presented as mean ± SEM. Student’s t-Test, Bonferroni correction for multiple comparisons.

Forced exercise has previously been shown to reduce formalin-induced spontaneous pain behavior [8]. We asked whether voluntary exercise (pre-training) similarly attenuates acute inflammatory pain responses. Formalin was administered approximately 16 hr after the final exercise session and spontaneous nocifensive behavior was recorded. Mice that received wheel access for 2 hr/night for 1–4 weeks displayed the same biphasic response to formalin as controls (Fig 3A–3C). To examine whether an increased dose of wheel running could affect acute inflammatory pain responses, exercise training was extended to 12 hr/night. On average mice ran 9.9 ± 1.2 km during 12 hr wheel running sessions. This distance is comparable to [17] if not greater than [13,29] distances reported by studies in which mice received home cage wheel access. Again, exercise for 2 weeks prior to testing did not influence formalin-induced spontaneous behavior (Fig 3D). Formalin injections took place 4–6 hr after completion of the final 12 hr/night exercise session. For each duration and dose of exercise tested, the cumulative time spent licking and lifting the hindpaw in the first (0–10 min) and second (11–60 min) phases of the formalin test were not different between exercise trained and control mice (Table 2).

**Table 2:** Cumulative time spent licking and lifting the hindpaw during Phases I and II of the formalin test.

	Phase I (0–10 min)		Phase II (11–60 min)	
Training	Control	Exercise	Control	Exercise
**1 week, 2hr/night**	129.3 ± 10.1s	110.9 ± 5.6s	296.2 ± 20.1s	275.4 ± 33.9s
**2 weeks, 2hr/night**	179.9 ± 17.9s	174.6 ± 16.8s	338.6 ± 47.1s	320.4 ± 30.7s
**4 weeks, 2hr/night**	91.9 ± 9.8s	105.7 ± 10.8s	338.9 ± 52.3s	281.8 ± 42.3s
**2 weeks, 12hr/night**	97.3 ± 15.5s	70.1 ± 23.0s	223.7 ± 49.8s	223.3 ± 46.9s

Data are presented as mean ± SEM, n = 4–6. Student’s t-Test, Bonferroni correction for multiple comparisons.

### Exercise training did not improve mechanical hypersensitivity or muscle wasting following peripheral nerve injury

The SNI rodent model of neuropathic pain produces prolonged and robust mechanical hypersensitivity in the ipsilateral hindpaw [39,49]. Forced exercise has been shown to reverse mechanical hypersensitivity in other peripheral nerve injury models including chronic constriction injury and spinal nerve ligation [9–11]. We asked whether voluntary wheel running could similarly reverse mechanical hypersensitivity caused by SNI.

**Fig 4:**
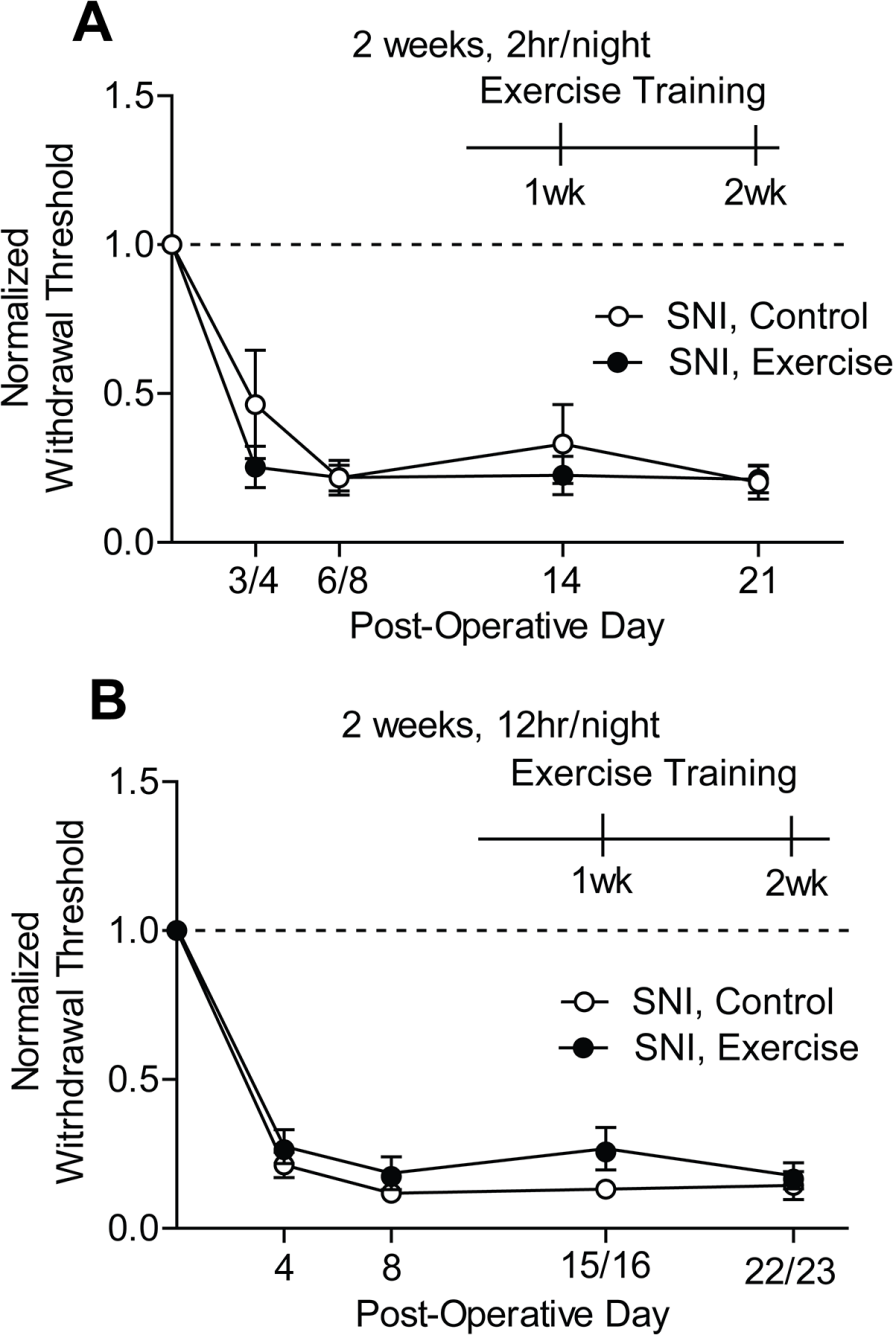
Exercise training did not improve SNI-induced mechanical hypersensitivity. **(A, B)** SNI gave rise to a significant reduction in mechanical withdrawal thresholds of the ipsilateral hindpaw in both SNI, control and SNI, exercise groups on POD 3/4 and 6/8 relative to baseline values (dotted line) (SNI, 2 hr/night p<0.0001, SNI, 12 hr/night p<0.0001, n = 7–8 and 5–7, respectively). When initiated 8 to 10 days post-op, 2 weeks of exercise training for either 2 hr **(A)** or 12hr **(B)** per night did not reverse SNI-induced mechanical hypersensitivity. Mechanical withdrawal thresholds were tested at one-week increments after exercise began. The one-week time point was on POD 14,15, or 16. The two-week time point was on POD 21, 22, or 23. Data are presented as mean ± SEM. Two-way RM ANOVA, Bonferroni correction for multiple comparisons.

Unilateral SNI produced a significant reduction in mechanical withdrawal thresholds relative to baseline (Two-way RM ANOVA: SNI, 2 hr/night p<0.0001, SNI, 12 hr/night p<0.0001) (Fig 4). Once the development of mechanical hypersensitivity was verified on post-operative days (POD) 3 or 4 and 6 or 8, we began exercise training mice on POD 8–10 to determine if voluntary exercise could reverse nerve injury-induced mechanical hypersensitivity. After each week of training, mechanical withdrawal thresholds were tested within 3 (12 hr/night group) to 12 hr (2 hr/night group) of the last training session. The one and two week post-training von Frey testing time points ranged from POD 14–16 and POD 21–23 across cohorts, respectively. Neither 2 hr/night (Fig 4A) nor 12 hr/night (Fig 4B) of exercise training for 2 weeks improved SNI-induced mechanical hypersensitivity.

The selection of a voluntary wheel running paradigm in the current study gave rise to the concern that nerve-injured mice would exercise much less, if at all, compared to uninjured mice. Such activity impairment would decrease the likelihood of observing exercise-induced improvements following SNI. However, quantification of weekly running distances indicates that SNI mice ran equivalent distances to uninjured animals each week of training when sessions were either 2 or 12 hr/night (Table 3). These findings eliminate concerns about significant differences in exercise dose between injured and uninjured mice.

**Table 3:** SNI did not depress weekly voluntary wheel running distances.

Training Session	2 hr/night		12 hr/night	
Mice	Week 1	Week 2	Week 1	Week 2
**Uninjured**	7.3 ± 0.5km	10.6 ± 0.9km	35.6 ± 5.2km	64.5 ± 6.5km
**SNI**	5.3 ± 0.3km	8.8 ± 0.5km	24.8 ± 2.4km	50.4 ± 6.2km

Data are presented as mean ± SEM. 2hr/night n = 8–34, 12hr/night n = 6–8. Student’s t-Test, Bonferroni correction for multiple comparisons.

**Fig 5:**
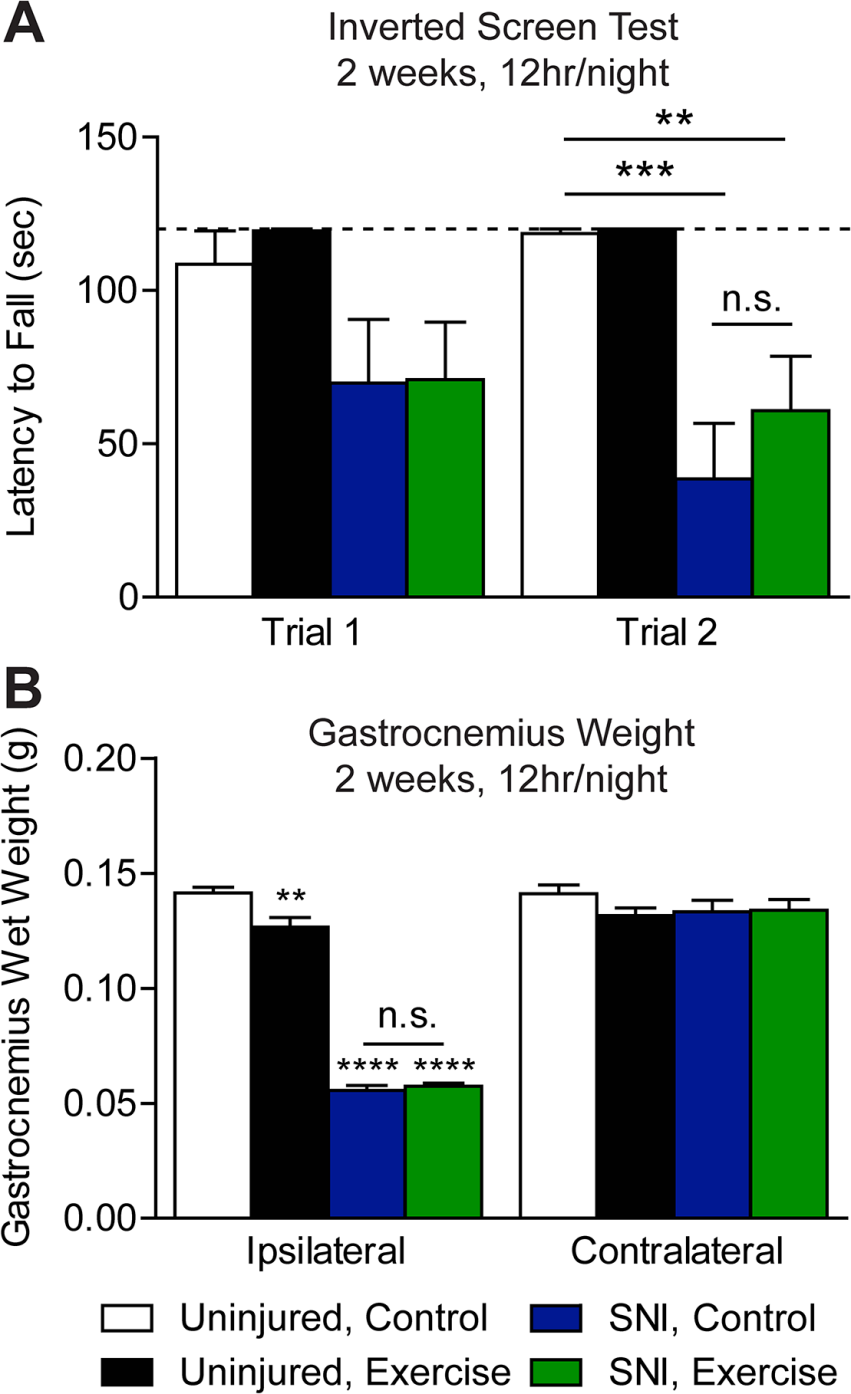
Exercise training did not reduce nerve injury-induced muscle wasting. **(A)** The inverted screen test of muscle strength was performed on mice 3 weeks (POD 21/22) after injury with a cut off latency of 120 sec (dotted line). SNI mice had a shorter latency to fall than uninjured mice in Trial 2 only (p = 0.0002). Despite exercise training for 12 hr/night for 9 nights, latency to fall of SNI, exercise mice was not improved compared to SNI, control mice, n = 6–8. **(B)** By POD 25 SNI gave rise to a robust reduction in ipsilateral gastrocnemius muscle wet weight (p<0.0001), which was unchanged by exercise, n = 6–8. Compared to uninjured controls, gastrocnemius muscle wet weight of uninjured, exercise mice was significantly reduced in the ipsilateral (p<0.01), but not the contralateral hindlimb. Data are presented as mean ± SEM. **p<0.01, ***p<0.001, ****p<0.0001, One-way ANOVA, Bonferroni correction for multiple comparisons.

In addition to hypersensitivity, sciatic nerve injury is associated with denervation, atrophy, and in turn muscle weakness [43,50]. Exercise following sciatic nerve injury can enhance muscle reinnervation, as well as grip strength and hindlimb motor function [10,21,51,52]. We tested if muscle strength is improved in SNI mice that were exercise trained for 12 hr/night for 2 weeks using the inverted screen test [42]. In trial one, both SNI, control and SNI, exercised mice had a shorter latency to fall than uninjured control mice that did not reach statistical significance (Fig 5A). In trial two, latency to fall of both SNI groups was significantly shorter than uninjured, control mice (One-way ANOVA: p = 0.0002). While mean latency to fall of SNI, exercise animals was longer than that of SNI, control animals, the difference is not statistically significant. Interestingly, neither SNI group exhibited a training effect between trials, which may be due to muscle atrophy. We found that SNI mice did not use the injured hindpaw to grip the screen. Consistent with this possibility, we did not find that muscle strength was improved following exercise training in uninjured animals. However, we used a cutoff latency of 120 sec, which may have masked potential effects of exercise on inverted screen performance in longer experimental trials. A previous study found that 8 weeks, but not 5 days, of home cage wheel access increases both forepaw and hindpaw grip strength in mice [13].

To test whether voluntary wheel running improved muscle atrophy following SNI, we measured gastrocnemius wet weight. On POD 25, we observed a significant reduction in ipsilateral gastrocnemius muscle wet weight in SNI mice compared to uninjured controls (One-way ANOVA: p<0.0001), while no change was observed in the contralateral muscle (Fig 5B). Wheel running for 2 weeks, 12 hr/night did not reduce gastrocnemius muscle wasting. We observed a significant reduction in wet weight of the ipsilateral (One-way ANOVA: p<0.01), but not contralateral gastrocnemius, of uninjured, exercised mice compared to uninjured controls (Fig 5B). A previous report indicates gastrocnemius wet weight is unchanged by forced or voluntary exercise in an uninjured context [23].

### Hindpaw epidermal innervation and thickness were not changed by exercise training in uninjured or nerve-injured animals

Voluntary wheel running has been shown to prevent increases in peptidergic IENF fiber density in a mouse model of prediabetic neuropathy [53]. We investigated whether wheel running changes IENF density or fiber phenotypic distribution in uninjured and SNI animals.

**Fig 6:**
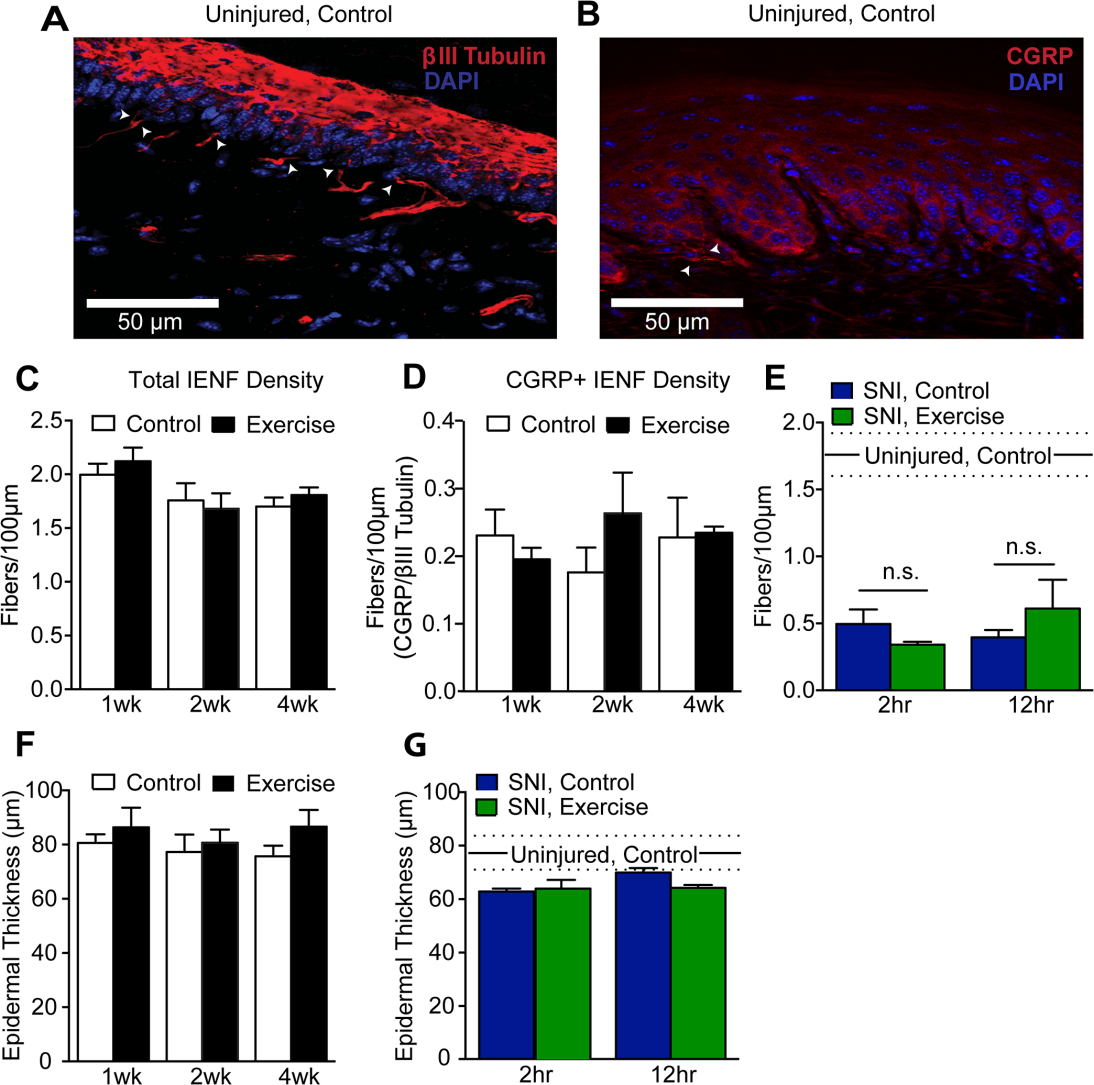
Exercise training did not alter hindpaw epidermal innervation or thickness in uninjured or SNI animals. **(A, B)** 30 μm sections of plantar hindpaw skin were stained with βIII tubulin (red) and CGRP (red) to quantify total and peptidergic IENF density, respectively. Arrowheads indicate IENF. DAPI (blue) staining was used to visualize the granular cell layer of the epidermis and measure epidermal thickness. **(C, D)** Both total (βIII tubulin+) and peptidergic (CGRP+) IENF density were unchanged by 1–4 weeks of exercise training in uninjured mice, n = 4–6. Student’s t-Test. **(E)** 25 days after injury, a significant reduction in total (βIII tubulin+) IENF density was observed in SNI, control mice compared to uninjured controls (p<0.0001, n = 4). Exercise training for either 2 or 12 hr per night for 2 weeks did not improve hindpaw epidermal innervation following SNI. One-way ANOVA. **(F)** Epidermal thickness was unchanged by 1–4 weeks of wheel running in uninjured mice, n = 4–6. Student’s t-Test. **(G)** Compared to uninjured controls, SNI, control hindpaw skin trended towards epidermal thinning that was unaffected by 2 or 12 hr/night of wheel running for 2 weeks, n = 4. One-way ANOVA. In panels E and G, mean and SEM of 2 week, 2 hr/night uninjured, controls are represented by sold and dashed lines, respectively. Data are presented as mean ± SEM. In all statistical analyses, a Bonferroni test was performed to correct for multiple comparisons.

To determine if exercise training alters total IENF density, peripheral nerve fibers in hindpaw skin sections were identified by the neuron-specific marker βIII tubulin (Fig 6A, red). Hindpaw innervation was further examined using a CGRP antibody as a marker of peptidergic fibers (Fig 6B, red). IENF were defined as those fibers crossing the dermal-epidermal border, which was visualized with DAPI (Fig 6A and 6B, blue). Quantification of total IENF density in hindpaw skin indicated no difference between control mice and mice exercise trained for 1–4 weeks, 2 hr/night (Fig 6C). The typically sparse CGRP+ IENF density relative to total IENF density was similarly unchanged by exercise at any time point (Fig 6D).

SNI causes marked denervation of the center aspect of plantar hindpaw skin within two weeks that begins to recover about 5 weeks after injury [54]. To determine if exercise reduces this denervation, center hindpaw total IENF density was quantified with βIII tubulin after two weeks of exercise, 25 days post-SNI. IENF density of SNI animals was significantly reduced compared to uninjured controls (One-way ANOVA: p<0.0001) (Fig 6E). However, wheel running for either 2 hr/night or 12 hr/night for 2 weeks did not improve IENF density of the ipsilateral hindpaw.

Intraepidermal fibers aid in the maintenance of the epidermis, and epidermal thinning is a consequence of denervation [54–57]. Therefore, epidermal thickness was also quantified alongside IENF density. Epidermal thickness was measured as the vertical distance from the dermal-epidermal border to the outermost granular cell layer. In uninjured mice, 1 to 4 weeks of exercise training did not give rise to changes in epidermal thickness (Fig 6F). In hindpaw skin of SNI, control mice, epidermal denervation was accompanied by epidermal thinning compared to uninjured controls, though the deficit did not reach statistical significance (Fig 6G). Exercise for 2 weeks did not prevent SNI-induced epidermal thinning. This result was expected as recovery of epidermal thickness after nerve injury is thought to be the product of reinnervation [55,58], and exercise did not reduce SNI-induced denervation.

## Discussion

We investigated the analgesic potential of voluntary wheel running in the contexts of acute inflammatory pain and neuropathic pain. We have shown that voluntary wheel running is not effective in attenuating formalin-induced nocifensive behavior or in improving SNI-induced mechanical hypersensitivity, muscle wasting, or skin denervation. We have also demonstrated that voluntary exercise training of uninjured animals does not cause changes in nociceptive thresholds, DRG excitability, or hindpaw innervation and epidermal thickness.

We report a lack of an effect of voluntary exercise training on nociceptive thresholds of uninjured animals. In contrast, Mathes *et al*. demonstrated significantly lower radiant heat tail flick response latencies, an end point we did not evaluate, following voluntary exercise in rats [59]. However, thermal and mechanical hindpaw withdrawal thresholds were unchanged in rats following forced swim training and in mice that had home cage running wheel access [13,14]. Our findings, along with previous studies reporting no effect of voluntary exercise on nociceptive thresholds in uninjured rodents, are consistent with human studies that have demonstrated basal pain thresholds are usually unchanged in athletes compared to non-athletes [60,61]. These data suggest that nociceptive thresholds are tightly regulated in an uninjured context to protect organisms from tissue damage without causing withdrawal from benign stimuli.

The finding that voluntary exercise training improves neither acute inflammatory pain responses nor recovery from peripheral nerve injury is in contrast to much of the existing literature. For instance, Kuphal *et al*. report that extended swimming for 9 days attenuates formalin-induced spontaneous pain [8]. Further, forced treadmill running has been shown to reverse thermal and mechanical hypersensitivity following both peripheral nerve and spinal cord injury [9–11,21,62].

A number of possible explanations may underlie our observation that exercise is not analgesic in the context of acute inflammatory and nerve injury-induced pain. Foremost, the current study differs from much of the existing literature due to the use of voluntary as opposed to forced exercise, which is reportedly more stressful [28,63]. For example, in a study conducted by Leasure and Jones, forced but not voluntary wheel running increased both anxiety-like behavior in the open field test and emotional defecation [23]. Moving animals in and out of running wheel cages is a potential source of stress. However, in our study, both control and exercise mice were transferred to running wheel cages during exercise periods. The two groups therefore differed only in exercise training because control mice were provided with locked running wheels. As training progressed, we observed noticeably fewer fecal pellets at the end of each session, suggesting that mice were not chronically stressed when placed into their individual cages. Importantly, cages of exercised mice were visibly cleaner than that of control mice, implying that voluntary exercise may have exerted an anxiolytic effect in our paradigm, as reported by others [31–33]. Collectively, our results indicate that the analgesic effect of exercise is potentially dependent on the nature of the exercise paradigm. Definitive proof that the different effects of voluntary versus forced exercise are related to stress will require additional investigation.

Another variable that could account for the differential effects of various exercise paradigms is exercise intensity. High intensity exercise has been shown to be more effective than low intensity exercise in reversing nerve injury-induced mechanical hypersensitivity in rats [9]. In our study, it is possible that voluntary wheel running is not of sufficient intensity to induce the physiological adaptions required to attenuate acute inflammatory pain or reverse SNI-induced mechanical hypersensitivity. However, voluntary wheel running has been shown to induce similar physiological adaptions as forced exercise paradigms that are effective in attenuating pain. Examples of these adaptions include increased expression of endogenous opioids and heat shock protein 72, as well as altered expression of growth factors [9,12,16,17,19,20,64–66]. In addition, one study found that wheel running of comparable distance to that observed in our study normalized behavioral hypersensitivity associated with pre-diabetic neuropathy [17]. These findings suggest that the exercise intensity of our paradigm is sufficient to engage the adaptive mechanisms contributing to exercise-induced analgesia. Other differences between our study and previous work such as social isolation and stress are more likely to contribute to the different outcomes.

Our study is the first to investigate the effects of exercise on SNI-induced neuropathic pain. While previous studies have shown that voluntary wheel running improves pain-like behavior in mouse models of chronic muscle pain and prediabetic neuropathy, voluntary wheel running failed to improve SNI-induced mechanical hypersensitivity in our hands [13,53]. Our results suggest that voluntary exercise-induced analgesia may be specific to certain types of neuropathies. The absence of exercise-induced improvements in our study may be due to the severe–and perhaps less modifiable–nature of SNI, in which ligation of the common peroneal and tibial nerves results in marked denervation of the hindpaw. Context-specific benefits of exercise have also been observed clinically. For example, aerobic exercise improves quality of life only in some type II diabetes patients, who often suffer from diabetic peripheral neuropathy [5,67]. It is also worth noting that clinically, improvements in quality of life do not necessitate improved pain ratings [68]. Rodent pain behavioral assays such as von Frey rely on nociceptive withdrawal thresholds as opposed to an endpoint that more comprehensiviely reflects a global pain experience. It is possible that voluntary exercise training can improve quality of life of nerve-injured mice. Efforts have been made to develop assays that more appropriately reflect clinical pain, but developing non-reflexive pain measures for rodent nerve injury models has proven to be difficult [69,70].

Another distinguishing quality of SNI is that after injury von Frey testing occurs on the lateral aspect of the hindpaw, which is solely innervated by the spared sural nerve [39]. Thus, we tested the sensitivity of uninjured fibers–though perhaps in an injured environment. Other rodent nerve injury models such as spinal nerve ligation and chronic constriction injury test hindpaw regions innervated by a combination of uninjured and injured afferents [46,47,71]. Therefore, it is possible that exercise preferentially attenuates injury-induced dysfunction in injured nerve fibers.

The timing of exercise intervention relative to nerve injury may also contribute to the lack of exercise-induced improvements following SNI. Because we were interested in whether exercise can reverse nerve-injury induced hypersensitivity, we chose to initiate exercise training on POD 8–10, once SNI-induced mechanical hypersensitivity was verified across multiple post-injury time points. In contrast, prior studies initiated forced exercise training by POD 7 [9–11,18]. In these studies, exercise either attenuated the development of or improved existing mechanical hypersensitivity caused by peripheral nerve injury. Therefore, it is possible that our voluntary wheel running paradigm could improve SNI-induced mechanical hypersensitivity if training began closer to the time of injury, i.e. within one week. However, Stagg *et al*. tested if timing of exercise onset after spinal nerve ligation determines the time required to reverse mechanical hypersensitivity [9]. When exercise was initiated either 1 or 4 weeks after injury, mechanical hypersensitivity was reduced within 3 weeks. The time at which exercise intervention must be initiated in order to recover nociceptive thresholds remains an interesting question.

Our study evaluated the benefit of exercise in the prevention of acute formalin-induced pain and in the reversal of nerve injury-induced chronic pain. We have shown that our exercise paradigm does not prevent formalin-induced inflammatory pain. However, it is possible that our exercise paradigm would prevent the development of chronic pain induced by nerve injury. Inflammation-induced pain and nerve injury-induced pain share a number of common mechanisms; however, there are important differences [72]. For instance, inflammatory pain is mediated through C fiber afferents, while neuropathic pain engages both C and Aβ fiber afferents [72]. In addition, spontaneous pain is mediated by C fibers [73]. It is unknown which fiber types are functionally influenced by voluntary wheel running in our study. Therefore, while our exercise paradigm did not prevent acute inflammation-induced spontaneous pain, it may prevent the development or delay the onset of SNI-induced mechanical hypersensitivity.

Lastly, it is also possible that exercise training of longer duration than our protocol of 2 weeks is needed to observe improvements of mechanical hypersensitivity following SNI. Duration of exercise training required to improve rodent pain-like symptoms varies in different studies. For instance, following chronic constriction injury of the mouse sciatic nerve, forced treadmill running within the first week of injury reduced mechanical hypersensitivity [10]. If treadmill running persisted for more than one week, however, improvements in mechanical thresholds were not observed. Similarly, in rats that underwent sciatic nerve transection, 2 weeks of treadmill running was sufficient to return mechanical thresholds to baseline [22]. However, in other studies, the effects of exercise training are not apparent unless exercise persists for periods longer than 2 weeks after injury. Reductions in mechanical and heat hypersensitivity caused by spinal nerve ligation in rats did not develop until after the third week of treadmill running [9]. Further, Groover *et al*. first observed reversal of prediabetic neuropathy-induced mechanical and visceral hypersensitivity after 8 weeks of voluntary wheel running [53].

The injury models examined in our study largely represent cutaneous pain. Even so, we cannot exclude injury to deep tissue or effects on deep tissue afferents (muscle) in either model. The SNI surgical procedure causes minimal damage to muscle; likewise, subcutaneous formalin injections could injure hindpaw muscle. However, the spontaneous nocifensive behavior resulting from subcutaneous formalin results from activation of cutaneous afferents and hindpaw von Frey is a measure of SNI-induced cutaneous hypersensitivity [38,74]. Our study therefore evaluates the effect of voluntary exercise on cutaneous pain. Measures of deep tissue pain in SNI mice after exercise training, though not within the scope of our study, would address whether voluntary exercise can reverse nerve injury-induced deep tissue pain, even though cutaneous hypersensitivity remains unchanged.

## Conclusions

The current study demonstrates that voluntary exercise training of uninjured animals does not alter nociceptive thresholds, sensory neuron excitability, or hindpaw intraepidermal innervation. We also demonstrate that voluntary wheel running fails to attenuate formalin-induced acute inflammatory pain and SNI-induced mechanical hypersensitivity, muscle wasting, and hindpaw denervation. When compared to existing literature, these results suggest that voluntary and forced exercise paradigms may have different analgesic potential. Further, the analgesic efficacy of voluntary exercise may be influenced by a number of variables including type of injury, timing of intervention, duration of exercise, and exercise intensity.
